# Supplementary material for: Performance benchmarking of LLMs on Chinese national medical licensing education: Cross-lingual and question-type effects
Source: PLoS One. 2026 Apr 8;21(4):e0346518. doi: 10.1371/journal.pone.0346518 (PMC13061252; doi:10.1371/journal.pone.0346518)
Supplement: Attachment — Submitted filename: Response to Reviewers.doc [file pone.0346518.s002.doc]

***Nanjing Medical University***

*Prof. Yuxia Tang*

Department of Radiology

*The First Affiliated Hospital with Nanjing Medical University*

*Nanjing 210029, P.R. China*

Dec 30, 2025

Dear Editor,

*PLOS ONE*

Thank you for your comments on our manuscript titled “Performance Benchmarking of LLMs on Chinese National Medical Licensing Educations: Cross-lingual and Question-Type Effects” (Manuscript Number: PONE-D-25-45754). We appreciate the editors and reviewers for devoting their time and effort to providing feedback and insightful comments. In accordance with the relevant suggestions, we have carefully responded to all the questions raised in the reviewers’ comments. Detailed corrections and responses are listed point by point below, and the corresponding revisions have been marked in red in the manuscript. We hope the revised manuscript meets the eligibility requirements for publication in the *PLOS ONE.*

**Reviewer #2**:

Reviewer #2: This paper discusses an interesting topic, but there are several issues that need to be addressed. If the authors resolve these concerns, the paper has the potential for publication.

1. The abstract mentions that the accuracy of large language models in answering questions from the Chinese Clinical Practice Physician Examination “has not been systematically evaluated.” In fact, there have been many studies in this area. It is suggested to modify this phrase to highlight that the comparison of cross-lingual and question-type effects has not been sufficiently explored, which emphasizes the significance of the current study.

**Our response:**

Thank you for this important observation. Accordingly, we have revised the statement in the Abstract as “The cross-lingual and question-type variations affecting large language models (LLMs) accuracy on the China's medical licensing educations remain insufficiently explored”. **(Page 2, Lines 2-4)**

2. In the abstract, it is stated that Doubao-1.5-pro and ChatGPT-4 achieved the highest accuracy. However, it is unclear whether this accuracy was observed for English or Chinese question types. This should be clarified.

**Our response:**

We thank the reviewer for this suggestion. We have revised the abstract as “Across all question types and languages, Doubao-1.5-pro achieved the highest accuracy at 92.0 % ± 1.3 %, whereas ChatGPT-4o had the lowest accuracy at 82.8 % ± 3.7 %”. **(Page 2, Lines 11-12)**

3. Please specify the source of the data used in the study. When referring to the “examination’s question bank,” is it an official source or from a third-party website? Also, which year's examination was used?

**Our response:**

We thank the reviewer for this suggestion. We have specified the data source in the Methods section as "The questions were curated from the official exam question pools spanning the years 2020 to 2022." **(Page 3, Lines 26-27)**

4. What is the total number of questions in the examination? The study used only 198 questions, how were these selected? Were they chosen randomly, or curated by clinians with some eligibility criteria? Additionally, do these questions include modalities beyond text, such as images, tables, or chemical formulas?

**Our response:**

We thank the reviewer for this important methodological point. The Chinese Medical Licensing Examination typically consists of 600 questions divided into three types (A, B, and C). From the official examination question bank, we employed a stratified random sampling approach to select questions proportionally from each question type. We randomly selected 198 paired English-Chinese questions with proportional representation from each question type category: 76 Type A items, 62 Type B items, and 60 Type C items. All questions were text-based single-choice questions without images, tables, or chemical formulas. **(Page 4, Lines 5-12)**

5. Please provide the specific dates when these models were used in the study, as well as their official release dates. This information will be useful for future comparative studies.

**Our response:**

We have included the study period and model release dates. We have revised the manuscript as “The study was conducted between May 13 to 20, 2025. The LLMs include ChatGPT-o3 (released April 17, 2025), ChatGPT-4o (released May 13, 2025), Gemini-2.5-pro (released March 25, 2025), Doubao-1.5-pro (released January 22, 2025), Deepseek R1 (released January 20, 2025), and Deepseek V3 (released December 26, 2024).” **(Page 4, Lines 1-3)**

6. The study mentions that the authors used web UI interfaces for the models. Could you provide more details on the model parameters, such as temperature, top-p, max tokens, etc.? These are typically default settings for large language models, and providing them would be helpful.

**Our response:**

We thank the reviewer for this valuable suggestion. In response, we have revised the methods as “All models were accessed through their official web interfaces using default parameters. Below are the input methods for different question types: for type A questions, each question was entered in a fresh conversation session; for type B questions, questions sharing the same stem were entered together in a single session, followed by a new conversation session for the next set of questions with a different shared stem. For type C questions, questions sharing the same set of options were entered together in one session, with a new conversation session initiated for the next group of questions with different shared options.”  **(Page 4, Lines 15-21)**

7. It would be beneficial to include more references that are closely related to the study, such as those with PMIDs 39729356 and 38355517.

**Our response:**

We thank the reviewer for this constructive suggestion. We have added the requested references. Added References:10. Zong H, Wu R, Cha J, Wang J, Wu E, Li J, Zhou Y, Zhang C, Feng W, Shen B. Large Language Models in Worldwide Medical Exams: Platform Development and Comprehensive Analysis. J Med Internet Res. 2024 Dec 27;26: e66114. doi: 10.2196/66114. PMID: 39729356. 11. Zong H, Li J, Wu E, Wu R, Lu J, Shen B. Performance of ChatGPT on Chinese national medical licensing examinations: a five-year examination evaluation study for physicians, pharmacists and nurses. BMC Med Educ. 2024 Feb 14;24(1):143. doi: 10.1186/s12909-024-05125-7. PMID: 38355517. **(Page 9, Lines 15-20)**

8. Is the passing standard for this exam 60% accuracy? If so, the authors should clarify in the discussion that the results of this study do not indicate that these large language models have passed the examination, since only 198 questions were used rather than the full set.

**Our response:**

We thank the reviewer for this constructive suggestion. In the revised Discussion section, we have expanded the Limitations subsection as “Although the passing standard for this examination is 60% accuracy, the results of this study do not indicate that these LLMs can pass the real examination. This is because the analysis was based on only 198 questions rather than the complete examination set, and it was limited to text-based single-choice questions, excluding items containing images, tables, chemical formulas, and other non-text modalities that are typically included in the full examination”. **(Page 7, Lines 14-19)**

9. The models provided explanatory rationales for their answers, but the authors only calculated the accuracy of the responses and did not perform a deeper analysis. For example, completeness, rationality, hallucinations, and reasoning behind the responses from the models were not discussed. This should be addressed in the discussion section.

**Our response:**

We thank the reviewer for this constructive suggestion. As the primary objective of this study was to systematically evaluate the cross-lingual and question-type effects on large language models' accuracy in answering questions from Chinese medical licensing examination, our analysis focused on quantifying performance differences across these dimensions. While we acknowledge that deeper analysis of the explanatory rationales—including completeness, rationality, hallucinations, and reasoning processes—would provide valuable insights, this was intentionally scoped as a future research direction rather than the primary focus of the current study. The complexity of medical reasoning explanations requires specialized evaluation frameworks that go beyond simple accuracy metrics. To address this concern, we have revised the manuscript and added a dedicated section in the Limitations as “This study only focused on accuracy, neglecting consistency, rationale, educational value, and clinical validity. Future studies are needed to address these issues.” **(Page 7, Lines 19-21)**

10. There are certain phrases, symbols, or text in the manuscript that may appear to be generated by generative AI. Could you clarify whether generative AI was used solely for text refinement, or if it was involved in other parts of the manuscript?

**Our response:**

We thank the reviewer for the careful review and valuable feedback. We appreciate your attention to detail regarding the potential use of generative AI in our manuscript. After careful review and revision of our manuscript, we confirm that no generative AI tools were used in the writing or content development of this study. All aspects of the manuscript—including conceptualization, methodology design, data analysis, interpretation of results, and writing of all sections—were conducted entirely by the human authors.
