# Supplementary material for: Performance benchmarking of LLMs on Chinese national medical licensing education: Cross-lingual and question-type effects
Source: PLoS One. 2026 Apr 8;21(4):e0346518. doi: 10.1371/journal.pone.0346518 (PMC13061252; doi:10.1371/journal.pone.0346518)
Supplement: Attachment — Submitted filename: Response letter-R1 2026.2.10.doc [file pone.0346518.s003.doc]

***Nanjing Medical University***

*Prof. Yuxia Tang*

Department of Radiology

*The First Affiliated Hospital with Nanjing Medical University*

*Nanjing 210029, P.R. China*

Feb 13, 2026

Dear Editor,

*PLOS ONE*

Thank you for your comments on our manuscript titled “Performance Benchmarking of LLMs on China's Medical Licensing Educations: Cross-lingual and Question-Type Effects” (Manuscript Number: PONE-D-25-45754R1). We appreciate the editors and reviewers for devoting their time and effort to providing feedback and insightful comments. In accordance with the relevant suggestions, we have carefully responded to all the questions raised in the reviewers’ comments. Detailed corrections and responses are listed point by point below, and the corresponding revisions have been marked in red in the manuscript. We hope the revised manuscript meets the eligibility requirements for publication in the *PLOS ONE.*

**Reviewer #1**:

1. *More detailed information about the Chinese National Physician Qualification Examination, including total number of questions, meaning of question types, subject distribution, how questions were obtained, why 2020–2022 were selected, why 198 pairs were sampled, and whether English translations were official.*

**Our response:** We thank the reviewer for this constructive suggestion. We agree that a more detailed description of the examination structure and methodology is essential for understanding the study’s validity. Accordingly, we have incorporated comprehensive information in the revised Methods section (**Page 3, Lines 26 to Page 4, Lines 20)**. **The Chinese medical licensing examination utilizes three standardized formats: Type A (independent items), Type B (shared-stem items), and Type C (shared-option items). While Type A items focus on foundational knowledge retrieval, Type B and Type C formats introduce significant contextual complexity. Type B items require the model to sustain clinical reasoning across a multi-stage vignette, while Type C items assess its precision in differential diagnosis when faced with a static set of competing distractors.**

Since official examination papers are not publicly released, all items for this study were sourced from widely recognized, publicly available preparatory materials provided by Beijing Medical Examination Assistance Technology Co., Ltd. We selected materials from 2020-2022 to ensure the inclusion of the most comprehensive and contemporary collections with verified answer keys. To ensure the statistical validity of this sample size, we calculated that 198 items drawn from an annual population of approximately 600 questions provide a margin of error of approximately ±5.8% at a 95% confidence interval (assuming a 50% response accuracy for maximum variance). This degree of precision is widely considered sufficient for the evaluative analysis of LLMs. To maintain consistency for cross-lingual evaluation, only text-based items were included, while questions containing images, tables, or chemical formulas were excluded. From this pool of available text-based items, we employed a stratified random sampling approach to select 198 questions, strictly preserving the original distribution of Type A, B, and C formats. This resulted in a dataset comprising 76 Type A, 62 Type B, and 60 Type C items.

The English versions were generated through a standardized forward-translation process conducted by two authors (YX Tang and SJ Wang), both of whom possess over ten years of clinical experience and have completed more than one year of formal medical training in the United States.

2. *This is not a prospective study but a cross-sectional study.*

**Our response:** We have replaced “prospective study” with “cross-sectional study” in both the Abstract sections. **(Page 2, Line 5)**

3. *Any questions that use figures/graphs/tables?*

**Our response:** We clarified that all included questions were text-only single-choice items, and that questions containing images, tables, or chemical formulas were excluded. This clarification has been added to the Methods section. **(Page 4, Lines 12-14)**

4. *The result should be compared with previous studies investigating license examinations in the same and different medical license examinations*

*doi: 10.2196/52784*

*doi: 10.2196/77978*

*doi: 10.2196/58897*

*doi: 10.1016/j.identj.2023.12.007*

**Our response:** We appreciate these helpful references. **The four prior studies evaluated either a single model or only a few models, focused on a single language, or did not distinguish question types, whereas our study simultaneously assesses six LLMs across two languages and three official question types.** We have incorporated all four studies into the Discussion, adding a new paragraph comparing our findings with prior evaluations of LLM performance on medical licensing examinations in China and internationally as “Our findings align with and extend previous research evaluating LLM performance on medical licensing examinations in China and internationally. Prior studies have shown that LLMs generally perform well on foundational recall questions but exhibit greater variability on complex reasoning tasks, consistent with our observation of significant question‑type effects [12, 14]. Other evaluations of Chinese medical licensing examinations similarly reported that English prompts may improve performance on basic knowledge tasks but offer limited benefit for higher‑order reasoning [15]. Additionally, recent work in dental and specialty examinations demonstrated that cross-lingual performance varies substantially across models, highlighting the importance of bilingual semantic alignment [16]. Our study contributes to this literature by providing a direct cross‑lingual comparison across three question types and identifying models with particularly strong cross‑lingual stability.” **(Page 5, Lines 19)**
